# Optimizing Vancomycin Soaking Protocols for Anterior Cruciate Ligament Reconstruction

**DOI:** 10.3390/antibiotics14040332

**Published:** 2025-03-22

**Authors:** Wachiraphan Parinyakhup, Piya Chavalparit, Dennapa Saeloh Sotthibandhu, Tanarat Boonriong, Korakot Maliwankul, Prapakorn Klabklay, Chaiwat Chuaychoosakoon

**Affiliations:** 1 Department of Orthopedics, Faculty of Medicine, Prince of Songkla University, Hat Yai 90110, Songkhla, Thailand; wachiraphun.p@psu.ac.th (W.P.); dartham@gmail.com (P.C.); tanarat.b@psu.ac.th (T.B.); drkorakot.m@gmail.com (K.M.); prapagorn.g@psu.ac.th (P.K.); 2 Faculty of Medical Technology, Prince of Songkla University, Hat Yai 90110, Songkhla, Thailand; dennapa.sa@psu.ac.th

**Keywords:** anterior cruciate ligament reconstruction, vancomycin soaking, bacterial eradication, infection prevention, cytotoxicity

## Abstract

**Background/Objectives**: Although current guidelines recommend soaking anterior cruciate ligament autografts in 5 mg/mL vancomycin for 20 min to reduce postoperative infections, practical constraints often limit soaking to 5 min. This study aimed to evaluate the bacterial eradication efficacy and cytotoxicity of various vancomycin concentrations and application methods within a 5 min soaking period. **Methods**: Human semitendinosus tendons were inoculated with one of four bacterial pathogens, including *Staphylococcus aureus*, *Staphylococcus epidermidis* with biofilm-producing and non-biofilm-producing strains, and *Enterococcus faecalis*. Samples were treated by direct soaking in 5 mg/mL vancomycin or gauze wrapping with 2.5, 5, or 10 mg/mL vancomycin for 5 min. Bacterial elimination was assessed using agar plating. Cytotoxicity toward human tenocytes and mesenchymal stem cells was evaluated at 6, 12, 24, and 72 h. Vancomycin release was measured using an immunofluorescence assay with the Cobas C311 Roche analyzer. **Results**: Complete bacterial eradication was achieved by direct soaking at 5 mg/mL and gauze wrapping at 10 mg/mL. All concentrations maintained cell viability above 70%, with no significant cytotoxicity. Vancomycin release was the highest in the direct soaking group, while it remained below the toxicity threshold for chondrocytes. **Conclusions**: Direct soaking at 5 mg/mL and gauze wrapping at 10 mg/mL for 5 min effectively eradicated bacterial contamination without compromising cell viability.

## 1. Introduction

Anterior cruciate ligament (ACL) injuries affect over 200,000 individuals annually in the United States, often necessitating surgical reconstruction to restore knee stability and function. Although ACL reconstruction is highly effective, it presents substantial risks, particularly postoperative infection, which remains a major complication in orthopedic surgery due to the susceptibility of the graft to bacterial inoculation.

Postoperative septic arthritis, a serious complication of ACL reconstruction, has been reported to occur at rates ranging from 0.2% [1] to 5.5% [2]. The most common causative organisms [3,4] include *Staphylococcus aureus* and coagulase-negative Staphylococci [3,4,5]. Management of these infections typically requires arthroscopic irrigation and debridement, with or without graft removal, followed by prolonged intravenous antibiotic therapy. These interventions impose physical and psychological burdens on patients, significantly impacting their quality of life and complicating recovery [6].

To address the risk of infection, multiple preventive strategies, including strict aseptic protocols, sterile techniques, and prophylactic antibiotics, are commonly employed [7]. Given that direct bacterial contamination of the ACL graft is a primary source of infection [8], pre-soaking autografts by wrapping them in gauze saturated with vancomycin solution, an antibiotic effective against common bacterial pathogens, has gained recognition as an adjunctive preventive measure. This approach has shown efficacy in reducing the incidence of postoperative septic arthritis, with current guidelines recommending soaking grafts in 5 mg/mL vancomycin solution for 20 min. However, practical constraints in clinical settings frequently limit the soaking time to 5 min [9]. In addition, some surgeons have adopted lower concentrations due to concerns about potential toxicity to intra-articular structures.

Despite the adoption of this method, postoperative infections persist. Potential factors contributing to suboptimal outcomes include reduced soaking durations, which may provide insufficient time for vancomycin to permeate fully; concentrations below the effective threshold for bacterial eradication; and limitations of the gauze-wrapping technique, in which vancomycin may not adequately penetrate into vulnerable regions of the graft, such as the center of a folded ACL graft. To address these concerns, the efficacy of a 5 min soaking protocol must be evaluated across different methods of vancomycin application.

Given that time constraints often necessitate shorter soaking durations, establishing whether a 5 min soak is sufficient to achieve effective bacterial eradication without inducing cytotoxic effects is essential for clinical applicability. The objectives of this study were to (1) evaluate the efficacy of bacterial eradication in hamstring grafts pre-soaked with vancomycin at varying concentrations, comparing the direct soaking method at a vancomycin concentration of 5 mg/mL with gauze wrapping methods at varying concentrations (2.5, 5, and 10 mg/mL) to identify the most effective method for enhancing bacterial eradication; (2) assess the cytotoxic effects on tenocytes and human umbilical cord-derived mesenchymal stem cells (HUMSCs) by measuring cell viability following exposure to vancomycin concentrations of 2.5, 5, and 10 mg/mL at 6, 12, 24, and 72 h; and (3) determine the vancomycin release profile from hamstring tendon autografts and compare these findings with established toxicity thresholds for osteoblasts and chondrocytes. We hypothesized that direct soaking in 5 mg/mL vancomycin would achieve superior bacterial eradication without tenocyte toxicity compared with the gauze-wrapping method.

## 2. Results

The microbiological effects of various concentrations of vancomycin on *S. aureus*, *S. epidermidis* (both biofilm-producing and non-biofilm-producing strains), and *E. faecalis* in contaminated tendon samples are summarized in Table 1. Complete bacterial eradication was achieved by directly soaking the samples in 5 mg/mL vancomycin and wrapping them in a gauze containing 10 mg/mL vancomycin.

**Figure 1:**
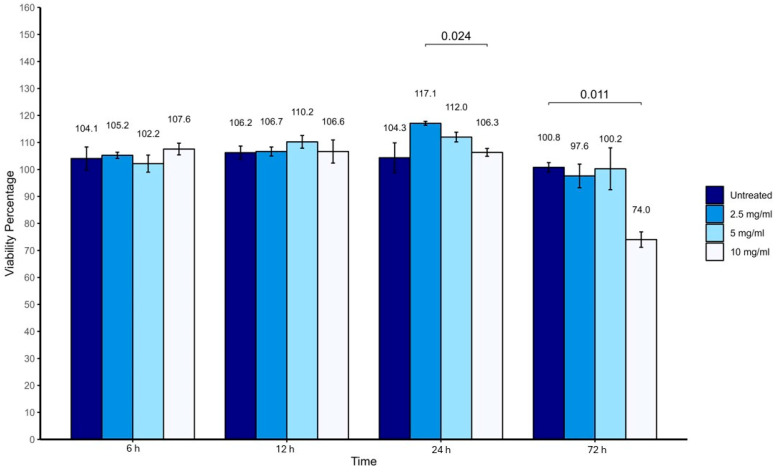
Percentage viability of human tenocytes after exposure to different concentrations of vancomycin for 6, 12, 24, and 72 h.

**Figure 2:**
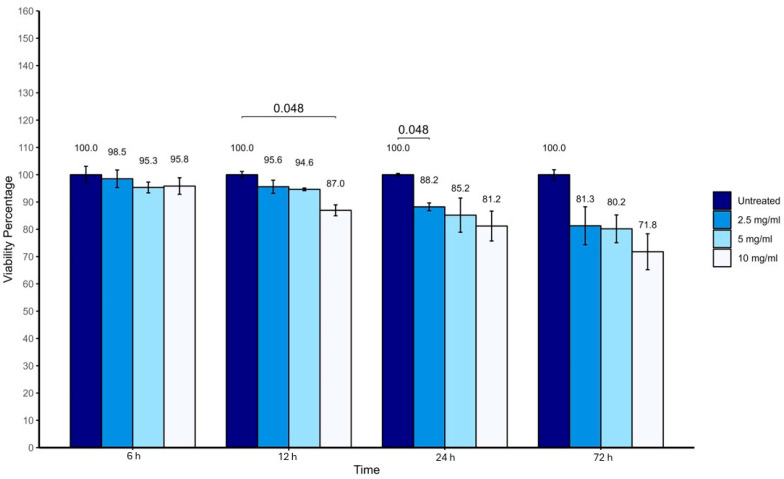
Percentage viability of human umbilical cord-derived mesenchymal stem cells (HUMSCs) after exposure to different concentrations of vancomycin for 6, 12, 24, and 72 h.

Vancomycin concentrations of 2.5, 5, and 10 mg/mL maintained high viability of tenocytes and HUMSCs at 6, 12, 24 and 72 h time points, as shown in Figure 1 and Figure 2. Tenocyte viability exceeded 100%, while HUMSC viability remained above 70% at all time points.

**Figure 3:**
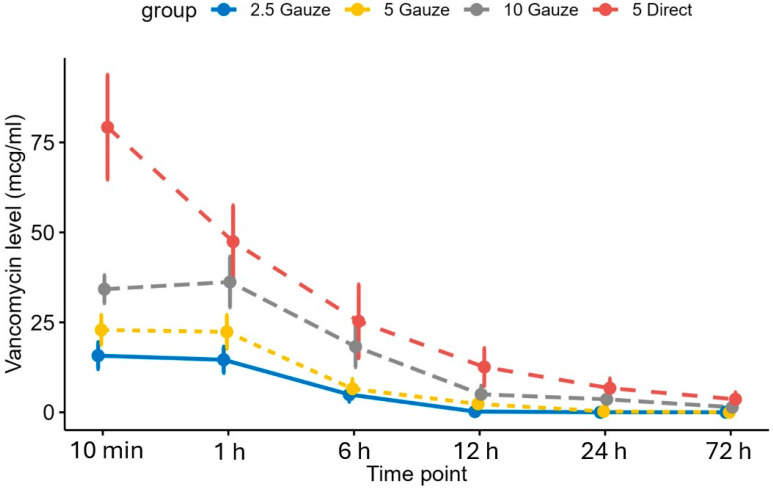
The average vancomycin concentrations in the phosphate-buffered saline solutions in all groups at each time point.

The average vancomycin concentrations in phosphate-buffered saline (PBS) across all groups at each time point are presented in Figure 3, which shows that the vancomycin release rate in all groups began to decrease after 1 h. The average vancomycin concentrations in the direct soaking group were significantly higher at all time points compared to the 2.5 and 5 mg/mL gauze-wrapping groups (*p* < 0.001). Additionally, while the concentrations in the direct soaking group exceeded those in the 10 mg/mL gauze-wrapping group at all time points, the difference was not statistically significant.

**Figure 4:**
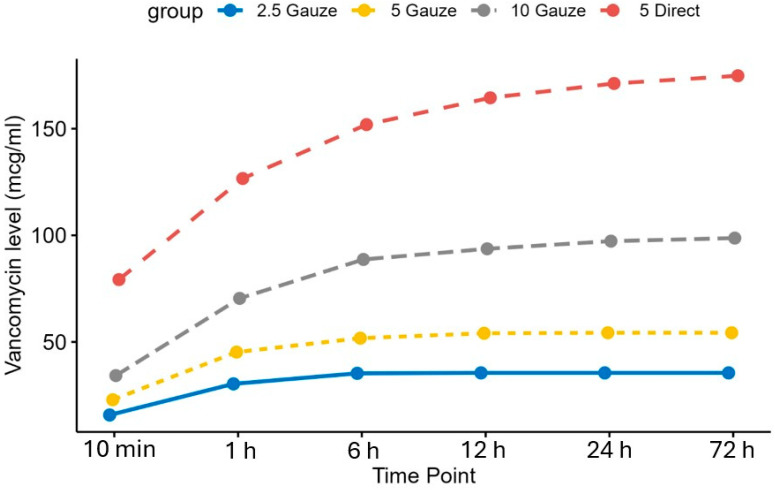
The cumulative doses of vancomycin released from the tendons in all groups.

The cumulative vancomycin release from tendons in each group is illustrated in Figure 4, showing that the direct soaking group demonstrated significantly greater release at all time points compared to the 2.5, 5, and 10 mg/mL gauze-wrapping groups (*p* < 0.05).

## 3. Discussion

Our study demonstrated that both the soaking method and vancomycin concentration significantly influence bacterial eradication and cellular safety. Specifically, we found that 10 mg/mL vancomycin with gauze wrapping and 5 mg/mL vancomycin via direct soaking for 5 min were effective for complete bacterial eradication. In contrast, 2.5 and 5 mg/mL vancomycin used with the gauze-wrapping method did not achieve complete bacterial eradication. Under all soaking conditions, tenocyte and HUMSC viability remained at or above 70%, indicating a favorable safety margin [10,11,12]. Furthermore, the vancomycin release levels remained well below the established toxicity thresholds for chondrocytes and osteoblasts, suggesting that these treatment protocols are within safe biological limits.

Postoperative infection following ACL adversely affects clinical outcomes and patient wellbeing. Infection rates have been reported to range from 0.2% [1] to 5.5% [2], with coagulase-negative Staphylococci, *S. aureus*, and Enterococci being the most implicated pathogens. Although current protocols for infection prevention recommend pre-soaking ACL grafts in gauze with a 5 mg/mL vancomycin solution for 20 min to mitigate the risk of infection, operating room time constraints often reduce the soaking duration to 10 or 15 min. While pre-soaking in vancomycin significantly decreases postoperative infection rates, complete eradication has not yet been achieved, with residual infection rates remaining above 0%. Studies employing lower vancomycin concentrations or shorter soaking durations have reported persistent infection rates. For example, Baron et al. [13] observed a 0.1% infection rate with a 10 min soak in a 1 mg/mL solution, while Carrozzo et al. [14] reported a 0.05% infection rate using a 2.5 mg/mL vancomycin solution for 10 min.

Residual infections may result from an insufficient vancomycin concentration for complete bacterial eradication or limited penetration of the vancomycin solution into regions prone to contamination, such as the center of the folded graft when gauze wrapping is used. Additionally, in cases where experienced surgeons perform ACL reconstructions with shorter operative times, the available soaking time may be limited to 5 min, which may be insufficient for complete bacterial eradication. In our study, a 5 min soaking duration was chosen to ensure applicability across all ACL reconstruction cases and alternative vancomycin concentrations; soaking methods were investigated within this timeframe. These results indicated that a concentration of 5 mg/mL with gauze wrapping for 5 min was insufficient for complete bacterial eradication. Based on these findings, we recommend either increasing the vancomycin concentration to 10 mg/mL with gauze wrapping for 5 min or employing direct soaking at 5 mg/mL for the same duration. Both methods appeared to enhance bactericidal efficacy while accommodating typical surgical time constraints.

An important consideration when soaking ACL grafts is ensuring bacterial eradication while avoiding any risk of intra-articular toxicity. To assess cellular toxicity in a direct exposure model, tenocytes and HUMSCs were cultured in vancomycin solutions at concentrations of 2.5, 5, and 10 mg/mL for 72 h. This approach simulated intra-articular conditions, allowing us to determine whether prolonged exposure to these concentrations compromised cell viability. Our findings showed that the viability of both cell types remained above 70%, indicating favorable biocompatibility. These results align with those of Rekowska et al. [12], who emphasized the importance of post-treatment processes to enhance the biocompatibility of photopolymerized poly(ethylene glycol) diacrylate materials, as residual unreacted components may otherwise reduce cell viability. Similarly, Podgórski et al. [11] underscored the importance of carefully validating cytotoxicity assays for nanofibrous materials due to assay-specific interactions. Teixeira et al. [10] reported that lactoferrin-functionalized lipid nanoparticles maintained cell viability above 70% at therapeutic concentrations, consistent with the cytotoxicity thresholds observed in our study. Collectively, these findings highlight the need to optimize both the concentration and exposure duration of vancomycin to balance its therapeutic efficacy with biocompatibility, particularly in sensitive cell populations such as mesenchymal stem cells. Notably, no cytotoxic effects were observed in either tenocytes or HUMSCs at any tested concentration, indicating that these vancomycin concentrations are safe under direct exposure conditions. While we acknowledge that the direct method may not fully replicate the dynamic environment in vivo, where drug concentrations decrease over time, our results suggest that cells would similarly tolerate lower, gradually diminishing concentrations in a clinical context.

To address this limitation and provide a more accurate toxicity assessment, we also employed an indirect method to measure vancomycin release levels from soaked grafts and compared these to the established toxicity thresholds for intra-articular cells. Our study demonstrated that the amount of vancomycin released from the grafts was influenced by three key factors: preparation method, vancomycin concentration, and immersion duration. Grafts wrapped in gauze soaked in higher vancomycin concentrations released greater quantities of the drug and sustained this release for longer periods than those treated with lower concentrations. As expected, semitendinosus tendons subjected to direct soaking released more vancomycin and maintained prolonged release times compared with those wrapped in gauze, even at equivalent vancomycin concentrations. Over the 72-h study period, the direct soaking method consistently released higher vancomycin levels than the gauze-wrapping method.

To address concerns related to cartilage toxicity, Shaw et al. [15] investigated the effects of vancomycin on chondrocyte viability using fresh osteochondral samples from juvenile porcine knee joints. Their study compared groups exposed to vancomycin concentrations of 2, 5, and 10 mg/mL with controls treated with saline. Chondrocyte viability was evaluated histologically using the Mankin criteria, and live/dead staining was performed using ethidium homodimer-2 and calcein AM. Confocal laser scanning microscopy revealed that vancomycin concentrations exceeding 5 mg/mL were cytotoxic to chondrocytes and osteoblasts. Similarly, Röhner et al. [16] examined the effects of vancomycin on primary human chondrocytes isolated from knee joint donors, using vancomycin at concentrations ranging from 0 to 50 mg/mL with exposure durations of up to 336 h. Cytotoxicity and cell viability were measured using lactate dehydrogenase and XTT enzyme-linked immunosorbent assays, while microscopy was used to assess cell integrity. The findings showed significant toxicity at vancomycin concentrations exceeding 12.5 mg/mL.

Given the variability in reported toxicity thresholds [15,16], we adopted a more conservative threshold of 5 mg/mL in our analysis to ensure safety. In our study, the highest average vancomycin concentration released at any time point across all preparation methods was 79.2 µg/mL, with the highest cumulative release reaching 174.81 µg/mL. Both values were well below the lowest toxicity threshold of 5 mg/mL for chondrocytes and osteoblasts, as established by Shaw et al. [15]. These findings suggest that the vancomycin concentrations used in our graft preparation methods pose minimal risk to chondrocyte and osteoblast viability.

This study had certain limitations. First, as an in vitro investigation, it might not have fully replicated the complex intra-articular environment, where factors such as blood flow, synovial fluid dynamics, and immune responses could affect vancomycin distribution and efficacy. Second, the direct cytotoxicity analysis was limited to human tenocytes and HUMSCs, which are representative intra-articular cells. Future research should expand testing to include additional relevant cell types, such as chondrocytes, osteoblasts, synoviocytes, and fibroblasts, to provide a more comprehensive assessment of the intra-articular safety of vancomycin. Additionally, subsequent studies should document the incidence of postoperative septic arthritis following ACL reconstruction using the proposed protocols, including gauze wrapping with 10 mg/mL vancomycin or direct soaking with 5 mg/mL vancomycin, and evaluate clinical outcomes to confirm the absence of toxicity in vivo. Third, we did not evaluate the mechanical properties of the grafts after vancomycin soaking. However, a previous study [17] examined soaking in 5 mg/mL vancomycin for 10 min and found no significant changes in graft properties. Future studies should investigate the mechanical effects of different vancomycin concentrations, soaking durations, and application methods to ensure graft integrity is maintained. Fourth, we did not directly compare the vancomycin concentrations used in this study with the systemic antibiotic levels typically achieved in joint fluid postoperatively. While our study measured vancomycin release from soaked grafts, further research is needed to compare these findings with systemic antibiotic pharmacokinetics to determine their clinical relevance. Fifth, the choice of gauze material could potentially affect drug absorption, release kinetics, and overall efficacy. Future studies should compare different gauze types to determine their influence on vancomycin delivery and bacterial eradication.

## 4. Materials and Methods

This in vitro study investigated bacterial eradication and absorption, as well as the release properties of vancomycin in human tendons, using various concentrations and preparation methods. This study was approved by the Ethics Committee of the Faculty of Medicine, Prince of Songkla University (REC 61-294-11-1, 67-493-11-1, 67-511-11-1, and 67-521-11-1).

Segments of the semitendinosus tendon measuring 0.5 × 0.5 cm^2^ and 1 × 1 cm^2^, obtained from unused surgical grafts, were used in phases 1 and 3 of the investigation, respectively. Graft diameters were measured using a Vernier caliper (Insize, Suzhou, China). To align with clinical practice, a 5 min soaking duration was adopted, as Pfeiffer et al. [9] identified this as the minimum feasible soaking time in ACL reconstruction settings.

### 4.1. Phase 1: Bacterial Eradication

#### 4.1.1. Bacterial Strains

The bacterial strains used in this study were *Staphylococcus aureus* ATCC 29213, *Staphylococcus epidermidis* ATCC 35984 (a biofilm-producing strain), *Staphylococcus epidermidis* ATCC 12228 (a non-biofilm-producing strain), and *Enterococcus faecalis* ATCC 29212. These pathogens are frequently associated with graft contamination and postoperative infections in ACL surgery, making them relevant for evaluating the efficacy of vancomycin. Each segment of the tendon graft was inoculated with exactly one bacterial strain.

#### 4.1.2. Bacterial Culture

The bacterial strains were cultured on tryptic soy agar and incubated at 37 °C overnight. Subsequently, 2–3 single bacterial colonies were selected and inoculated in tryptic soy broth, followed by incubation at 37 °C for 4 to 6 h. The bacterial suspensions were adjusted to a turbidity standard of 0.5 McFarland, corresponding to a concentration of 1.5 × 10^8^ colony-forming units (CFU)/mL, using a sterile 0.85% saline solution. The bacterial suspension was diluted in saline to a concentration of 1 × 10^4^ CFU/mL for experimental use.

#### 4.1.3. Bacterial Elimination Using Vancomycin

A total of 20 tendon segments per group, each measuring 0.5 × 0.5 cm^2^, were immersed in bacterial suspensions for 30 min. Tendon segments were then randomly assigned to four groups of 20 samples each and treated with vancomycin. In three of these groups, the tendons were wrapped with sterile gauze soaked in vancomycin at concentrations of 5, 10, and 20 mg/mL for 5 min. In the fourth group, the tendons were directly immersed in a 5 mg/mL vancomycin solution for 5 min. A control group was treated with sterile saline. Afterward, the tendons were rolled onto nutrient agar plates and incubated at 37 °C for 24 h to evaluate bacterial growth.

### 4.2. Phase 2: Evaluating Cytotoxicity on Tenocytes and HUMSCs

Primary tenocytes were isolated from human semitendinosus tendons and cultured in a humidified incubator at 37 °C with 5% CO_2_. Tenocytes were chosen because they represent the primary cell type in tendon tissue, reflecting potential intra-articular toxicity to the graft itself. To grow primary tenocytes, tendons were cut into small pieces and placed in Dulbecco’s Modified Eagle’s Medium, a high-glucose medium, supplemented with 20% fetal bovine serum and 1% penicillin–streptomycin. After 2 weeks, tenocytes were observed. Cells were maintained until they reached confluency and were subsequently subcultured using trypsin-ethylenediaminetetraacetic acid. Cells from passages three and four were used in subsequent experiments. No phenotypic variation was observed.

HUMSCs were cultured in Dulbecco’s Modified Eagle’s Medium, a high-glucose medium, supplemented with 20% fetal bovine serum and 1% penicillin–streptomycin. The cultures were maintained at 37 °C under 5% CO_2_ with humidity. The medium was replaced every 3 to 4 days, and cells were cultured until they reached the appropriate confluency before further use. HUMSCs were included to model multipotent cells that could be present intra-articularly or in synovial fluid.

To evaluate cell viability in response to vancomycin exposure, tenocytes and HUMSCs were treated with vancomycin at concentrations of 5, 10, and 20 mg/mL for 6, 12, 24, and 72 h. Cell viability was assessed using the 3-(4,5-dimethylthiazol-2-yl)-2,5-diphenyltetrazolium bromide (MTT) assay. Each cell type was seeded in 96-well plates at a density of 5 × 10^3^ cells per well with 200 µL of medium. Cells were plated 2 days before the experimental procedures. Once the cells adhered to the bottom of the wells, vancomycin at specified concentrations was added. After the designated incubation period, the medium was aspirated and fresh medium supplemented with sterile-filtered MTT was added to each well. After 4 h of incubation, the solutions were removed, and dimethyl sulfoxide was added to dissolve the formazan crystals. The plates were subsequently incubated for 10 min to ensure complete dissolution. Absorbance was measured at 540 nm using a microplate reader. Cell viability was calculated as a percentage using the following formula:

Percentage of cell viability = (OD570 nm-treated cells/OD570 nm-untreated cells) × 100

The experiment was performed in triplicate.

### 4.3. Phase 3: Vancomycin Release from Hamstring Autograft

Vancomycin release levels were determined at various time intervals using an immunofluorescence assay with the Cobas C311 Roche analyzer (Roche Diagnostics, Mannheim, Germany). A total of 88 semitendinosus tendon grafts were randomly assigned to four groups (*n* = 22 per group). Samples in the first three groups were wrapped in 2 × 2 cm gauze squares pre-soaked for 5 min in vancomycin solutions at concentrations of 2.5, 5, and 10 mg/mL, respectively. The fourth group consisted of grafts directly soaked in a 5 mg/mL vancomycin solution. Following the initial 5 min soak, all samples were transferred to 4 mL of PBS (pH 7.4) for 10 min to assess the initial vancomycin release. This procedure was repeated at cumulative time intervals of 1, 6, 12, 24, and 72 h to construct the experimental timeline. Data on graft diameter, donor graft sex, and vancomycin levels at each time point were recorded. The cumulative vancomycin release was calculated.

### 4.4. Method Exploration

The graft-soaking methods were categorized into four groups based on common clinical practices, using 5 mg/mL as the baseline vancomycin concentration [17]. To assess the safety of the four preparation methods, the released vancomycin concentrations were analyzed and compared to established toxicity thresholds for chondrocytes and osteoblasts. Previous studies have identified 5000 mcg/mL as the upper safety limit [15,16].

### 4.5. Timing of Vancomycin Release Evaluation

The timing for the evaluation of vancomycin release was selected to capture both initial and sustained release dynamics. Samples were initially immersed in PBS for 10 min to allow vancomycin diffusion and establish a baseline antibiotic concentration. Subsequent measurements were taken at 1, 6, 12, 24, and 72 h to monitor ongoing release. This approach enabled a comprehensive evaluation of antibiotic availability post-implantation, beginning with immediate-release kinetics.

### 4.6. Statistical Analysis

All statistical analyses were performed using R software (version 4.1.1) with the “epicalc” package (R Foundation for Statistical Computing, Vienna, Austria). Bacterial eradication rates and cell viability were quantified as percentages. Differences in vancomycin release among the four preparation methods were analyzed using a one-way analysis of variance, followed by Holm’s correction to adjust for multiple comparisons. Subsequent pairwise comparisons between preparation methods were performed using Student’s *t*-test. The associations were considered statistically significant at *p*-values < 0.05.

## 5. Conclusions

In conclusion, this study demonstrated that soaking ACL grafts in vancomycin, either through gauze wrapping at 10 mg/mL or direct soaking at 5 mg/mL for 5 min, effectively eradicated bacterial contamination without inducing cytotoxicity in key intra-articular cell types. While the in vitro results indicate that both methods are safe and effective under controlled conditions, further in vivo studies are recommended to validate these findings in clinical settings.

## Figures and Tables

**Table 1:** Bacterial culture outcomes under varying vancomycin concentrations and application methods.

Group	*Staphylococcus aureus* ATCC 29213 (*n* = 20)		*Staphylococcus epidermidis* ATCC 12228 (*n* = 20)		*Staphylococcus epidermidis* ATCC 35984 (*n* = 20)		*Enterococcus faecalis* ATCC 29212 (*n* = 20)	
	Positive Culture	Percentage	Positive Culture	Percentage	Positive Culture	Percentage	Positive Culture	Percentage
2.5 mg/mL (gauze wrapping)	3	15	10	50	4	20	7	35
5 mg/mL (gauze wrapping)	0	0	0	0	1	5	0	0
10 mg/mL (gauze wrapping)	0	0	0	0	0	0	0	0
5 mg/mL (direct soaking)	0	0	0	0	0	0	0	0
Control (NaCl)	20	100	20	100	20	100	20	100

## Data Availability

The original contributions presented in this study are included in the article. Further inquiries can be directed to the corresponding author.

## Abbreviations

The following abbreviations are used in this manuscript:

ACL	anterior cruciate ligament
ATCC	American Type Culture Collection
CFU	colony-forming unit
HUMSC	human umbilical cord-derived mesenchymal stem cell
MTT	3-(4,5-dimethylthiazol-2-yl)-2,5-diphenyltetrazolium bromide
PBS	phosphate-buffered saline
